# The impact of metabolic dysfunction-associated steatotic liver disease on the efficacy of pegylated interferon-based therapy in patients with chronic hepatitis B

**DOI:** 10.3389/fimmu.2025.1708909

**Published:** 2026-01-09

**Authors:** Jiaxin Han, Lianxin Xu, Yuqin Li, Ziling Wang, Minghui Zeng, Jieru Gao, Yuqiang Mi, Wentao Kuai, Huiling Xiang, Liang Xu

**Affiliations:** 1 The Second People’s Hospital Affiliated to Tianjin Medical University, Tianjin, China; 2 Tianjin Institute of Hepatology, Tianjin Second People’s Hospital, Tianjin, China; 3 Department of Hepatology & Oncology, Tianjin Second People’s Hospital, Tianjin, China; 4 Endoscopy Center, Tianjin Second People’s Hospital, Tianjin, China; 5 Department of Infectious Diseases, Tianjin Fourth Central Hospital, The Affiliated Hospital of Tianjin Medical University, Tianjin, China; 6 Department of Gastroenterology and Hepatology, Tianjin University Central Hospital (The Third Central Hospital of Tianjin), Tianjin, China

**Keywords:** metabolic dysfunction-associated steatotic liver disease, chronic hepatitis B, pegylated interferon, efficacy, outcome events

## Abstract

**Introduction:**

The impact of metabolic dysfunction-associated steatotic liver disease (MASLD) on the efficacy and prognosis of pegylated interferon (Peg-IFN)-based therapy for chronic hepatitis B (CHB) remains controversial and requires further investigation.

**Methods:**

A total of 620 CHB patients receiving Peg-IFN-based therapy were enrolled and categorized into MASLD-CHB (n = 247) and CHB (n = 373) groups. Propensity score matching (PSM) was employed to balance baseline differences. Kaplan-Meier survival analysis and LASSO-Cox regression were applied to identify independent predictors of complete virological response (CVR) and hepatitis B surface antigen (HBsAg) seroclearance.

**Results:**

After PSM, 247 patients were included in each group. Compared with the MASLD-CHB group, the CHB group exhibited significantly higher cumulative CVR rates at month 3 (36.84% *vs.* 24.54%, *p* = 0.041), month 12 (77.19% *vs*. 62.72%, *p* = 0.013), end of treatment (EOT) (78.94% *vs*. 65.45%, *p* = 0.019), and end of follow-up (EOF) (80.70% *vs*. 68.18%, *p* = 0.026). The median time to the first CVR was shorter in the CHB group (4.4 *vs*. 5.5 months, *p* = 0.030). LASSO-Cox regression revealed HBV DNA (HR: 0.486, 95% CI: 0.297-0.793, *p* = 0.004), MASLD (HR: 0.736, 95% CI: 0.550-0.986, *p* = 0.040), HBsAg (HR: 0.808, 95% CI: 0.706-0.926, *p* = 0.002), HBeAg positivity (HR: 0.611, 95% CI: 0.433-0.862, *p* = 0.005), and triglycerides (HR: 0.793, 95% CI: 0.661-0.951, *p* = 0.012) as independent suppressors of CVR. Multiple factors were associated with HBsAg seroclearance (*p* < 0.05). At the EOF, the MASLD-CHB group had higher liver stiffness measurement values (8.10 [6.40, 9.60] *vs*. 6.20 [5.00, 7.50]) and a greater proportion of moderate-to-severe fibrosis (18.7% *vs*. 5.9%, *p* < 0.01).

**Conclusion:**

In CHB patients receiving Peg-IFN-based therapy, concurrent MASLD may impede the reduction of HBV DNA levels, delay the time of first CVR, and potentially accelerate liver fibrosis progression.

## 1 Introduction

The implementation of preventive measures, such as the hepatitis B virus (HBV) vaccine and strategies to interrupt mother-to-child transmission, has significantly reduced the incidence of new chronic hepatitis B (CHB) cases (1). Nevertheless, approximately 292 million individuals worldwide remain affected, making CHB a serious global health concern, particularly in low- and middle-income countries (2). CHB is a leading cause of cirrhosis and hepatocellular carcinoma (HCC), responsible for 500,000 to 1.2 million deaths annually, with nearly 60% attributed to HCC (3). Meanwhile, lifestyle changes have driven non-alcoholic fatty liver disease (NAFLD) to become the most prevalent chronic liver disease globally, posing a major public health threat (4). The global prevalence of NAFLD is approximately 32.4% and has increased significantly over time (5). Both CHB and NAFLD are common etiologies of chronic liver disease worldwide, and their concurrent presence is increasingly observed in clinical practice (6). Studies indicate that 15%-30% of CHB patients also have NAFLD, and those with both conditions tend to experience more rapid disease progression (7, 8). In 2023, Rienstra et al. reached a consensus through a four-round Delphi survey (9), renaming NAFLD as metabolic dysfunction-associated steatotic liver disease (MASLD).

The relationship, interaction, and underlying mechanisms between CHB and MASLD remain unclear, making it a key focus in liver disease research. Some studies suggest that hepatic steatosis in CHB patients is primarily driven by host metabolic factors, while MASLD progression may activate immune cells and metabolic components, directly suppressing HBV replication or indirectly inducing antiviral immune responses, thereby exerting beneficial effects in CHB patients (6). However, existing findings on virological and biochemical responses have been inconsistent. The impact of concurrent MASLD on CHB treatment efficacy or prognosis remains controversial. Furthermore, most related studies have focused on nucleos(t)ide analogues (NAs) antiviral therapy, with limited data available on pegylated interferon (Peg-IFN)-based treatment (10–12). Therefore, clarifying the impact of concurrent MASLD on antiviral response, biochemical parameters, lipid metabolism, and clinical outcomes in CHB patients receiving Peg-IFN-based therapy is crucial for optimizing clinical management and improving prognosis.

## 2 Materials and methods

### 2.1 Study population

**Figure 1:**
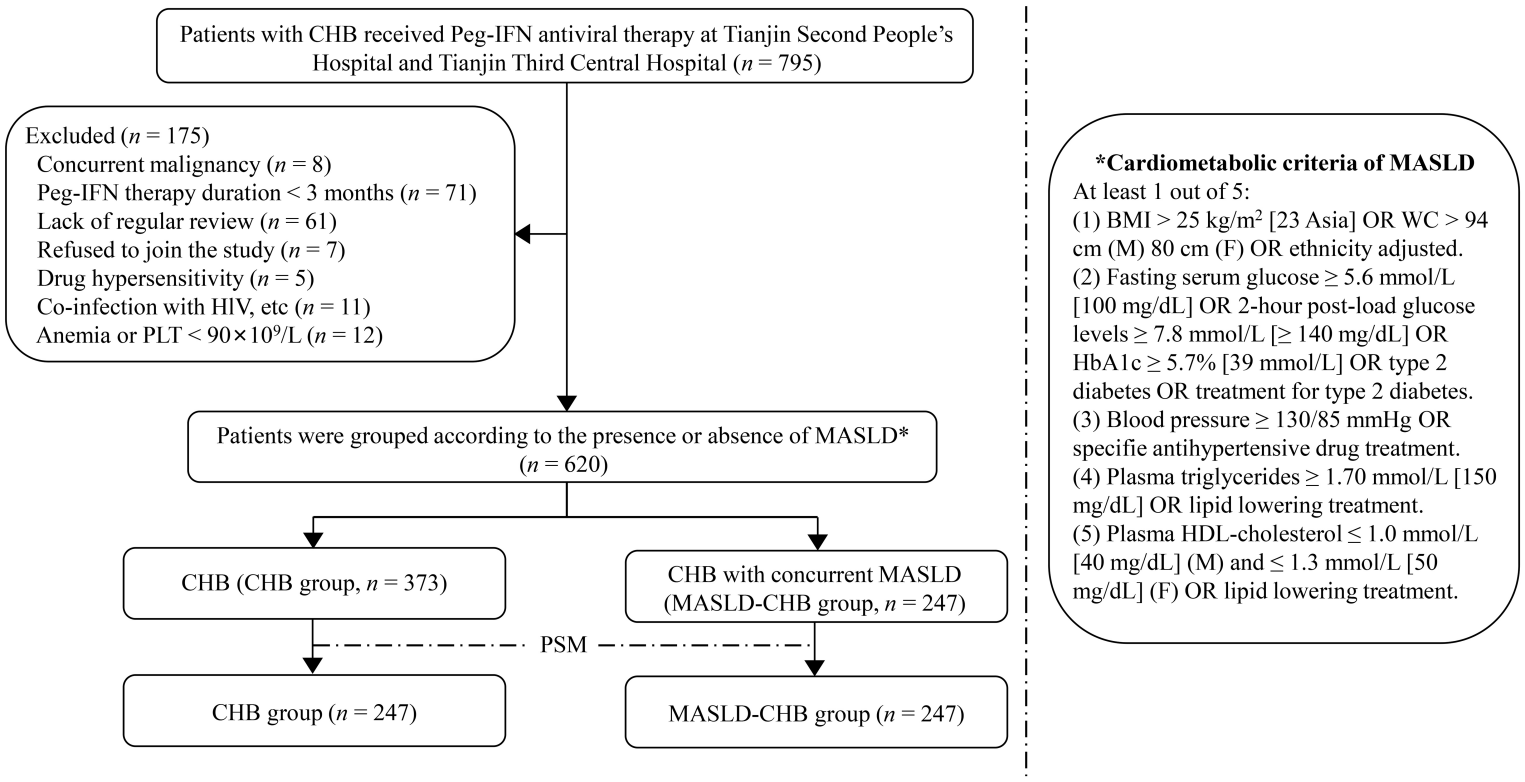
Flow diagram of patient enrollment in this study.

This study included 620 CHB patients receiving Peg-IFN-based therapy at Tianjin Second People’s Hospital and Tianjin Third Central Hospital from July 2020 to December 2024, according to the inclusion and exclusion criteria. Patients were classified into two groups: the CHB group (n=373) and the MASLD-CHB group (n=247), according to the presence or absence of MASLD. **Figure 1** provides a detailed flowchart of patient inclusion in this study. The study protocol was approved by the Medical Ethics Committee of Tianjin Second People’s Hospital and adheres to the principles of the Declaration of Helsinki (13), as well as the ethical requirements for research involving human subjects.

### 2.2 Inclusion and exclusion criteria

Inclusion Criteria: (1) Participants aged 18–65 years; (2) for women of childbearing age, a negative urine or blood pregnancy test is required before enrollment; (3) patients with persistent hepatitis B surface antigen (HBsAg) positivity for ≥ 6 months, meeting the diagnostic criteria for CHB as outlined in the EASL Clinical Practice Guidelines on the management of hepatitis B virus infection (14); (4) patients who have received at least 3 months of regular Peg-IFN therapy; (5) for CHB patients with concurrent MASLD, abdominal ultrasound must demonstrate evidence of fatty liver and meet the diagnostic criteria for MASLD as defined in the EASL-EASD-EASO Clinical Practice Guidelines on the management of metabolic-associated fatty liver disease (15); (6) complete general, clinical, and follow-up data available and (7) willingness to undergo treatment and sign informed consent. Exclusion Criteria: (1) Patients with concurrent liver tumors, malignant hematologic diseases, or other malignancies, or those who have received antitumor or immunomodulatory treatment within 6 months prior to enrollment; (2) patients allergic to Peg-IFN; (3) patients with a history of organ transplantation or immunosuppressive therapy, and (4) those with concurrent infections of hepatitis C virus, hepatitis D virus, or human immunodeficiency virus.

### 2.3 Data collection

Patient information, including gender, age, height, weight, comorbidities (diabetes, hypertension), alcohol consumption history, history of antiviral treatment, and relevant clinical outcomes, was collected via the electronic medical record system. Body mass index (BMI) was calculated. Biochemical parameters, including alanine aminotransferase (ALT), aspartate aminotransferase (AST), gamma-glutamyl transferase (GGT), alkaline phosphatase (ALP), total bilirubin (TBIL), creatinine (Cr), total cholesterol (TC), triglycerides (TG), high-density lipoprotein (HDL), low-density lipoprotein (LDL), and glucose (GLU), were measured using the HITACHI 7180 automated biochemical analyzer (Japan). Routine blood tests, including white blood cell (WBC) count, neutrophils (NEUT), red blood cell (RBC) count, hemoglobin (HGB), and platelets (PLT), were performed using the Sysmex XN-2000 automated blood analyzer (Japan). Hepatitis B serological markers, including HBsAg, hepatitis B surface antibody (HBsAb), hepatitis B e antigen (HBeAg), hepatitis B e antibody (HBeAb), and hepatitis B core antibody (HBcAb), were measured using the Roche COBAS e411 electrochemiluminescence immunoassay analyzer (68305 Mannheim, Germany). HBV DNA quantification was performed using real-time quantitative polymerase chain reaction (PCR) technology, with a lower limit of detection of 20 IU/mL; results were log-transformed to log_10_ IU/mL. Tumor markers, such as alpha-fetoprotein (AFP), were assessed using enzyme-linked immunosorbent assay (ELISA).

Liver stiffness measurement (LSM) and controlled attenuation parameter (CAP) were assessed using the FibroScan^®^ 502 (Echosens, France) by trained specialists. Prior to the measurement, patients were instructed to fast for 4–6 hours, lie in a supine position, and elevate the right arm above their head to increase the intercostal space. Measurement points were selected at the 7th or 8th intercostal space along the right anterior axillary line. The probe was placed perpendicular to the measurement site, with the depth set to 4–5 cm beneath the liver capsule. Ten valid measurements were taken, with at least 60% of the total measurements deemed valid. The median value of the valid measurements was selected as the result, and the interquartile range (IQR) to median ratio (IQR/med) was required to be < 30%, with a success rate (successful measurements/total measurements) ≥ 60% (16).

Liver ultrasound was performed using the ultrasound (PHILIPS, IU22-22100, US) with a 3.5 MHz probe, operated by experienced sonographers. Patients were instructed to fast for at least 8 hours and positioned supine with the abdomen fully exposed. Following the application of a coupling agent to the probe, the liver was scanned in multiple planes to assess its size, shape, echogenicity, and the presence of abnormal echo areas (17).

### 2.4 Medication and follow-up

All patients received Peg-IFN alpha-2b injection (Peg-IFN α-2b, 135 μg/180 μg, S.C., QW) as the primary antiviral therapy. In the event of moderate to severe adverse reactions requiring dose adjustment during treatment, the dose was initially reduced to 135 μg. Once the adverse reactions resolved, the dose could be restored to the initial level as appropriate. Dose reduction should be considered if the NEUT < 1.5×10^9^/L or PLT < 50×10^9^/L. Discontinuation of the medication is recommended if the NEUT < 0.5×10^9^/L or PLT < 25×10^9^/L (18). For patients who achieved HBsAg seroclearance, the decision to continue consolidation therapy for 3–6 months would be made by the physician, based on the patient’s clinical condition and preferences, with HBsAg seroconversion as the ideal treatment endpoint.

This study included patients who received Peg-IFN for ≥ 3 months and had a follow-up period of ≥ 18 months. Follow-up assessments were conducted at key time points: baseline at the initiation of Peg-IFN-based therapy, and at the 3rd, 6th, and 12th months, as well as at the end of treatment (EOT) and end of follow-up (EOF). Considering differences in adherence, a ±4-week window for follow-up was allowed, with the follow-up period concluding in June 2025.

### 2.5 Efficacy and definitions

In this study, complete virological response (CVR) was defined as a reduction in HBV DNA to below the detection limit (< 20 IU/mL) following Peg-IFN-based therapy. HBsAg seroclearance was defined as HBsAg-negative, with or without HBsAb-positive. HBsAg seroconversion was defined as HBsAg-negative with HBsAb-positive. HBeAg seroclearance was defined as HBeAg-negative, with or without HBeAb-positive. HBeAg seroconversion was defined as HBeAg-negative with HBeAb-positive (19).

### 2.6 Statistical analysis

Statistical analyses were conducted using R version 4.4.1, GraphPad Prism 10, and SPSS 28.0. Categorical variables were described as frequencies (percentages), and group comparisons were performed using the Chi-squared (χ²) test or Fisher’s exact test. The normality of continuous variables was assessed using the Shapiro-Wilk test. Normally distributed continuous variables were expressed as means ± standard deviations (X ± S), and intergroup comparisons were performed using independent-sample t-tests. Non-normally distributed data were presented as medians (IQR 25-75%) and compared between groups using the Mann-Whitney *U* test or Kruskal-Wallis *H* test. A p-value of < 0.05 was considered statistically significant. Propensity score matching (PSM) with a 1:1 ratio was used to balance differences between the groups, with a caliper value set at 0.1. Kaplan-Meier (K-M) survival analysis was used to assess CVR and HBsAg seroclearance between the two groups, with intergroup comparisons conducted using the log-rank test.

Univariate Cox regression analysis was performed to investigate independent factors associated with CVR and HBsAg seroclearance. Variables with a p-value < 0.10 were included in LASSO regression to address multicollinearity, and cross-validation was used to determine the optimal λ value. Non-zero coefficients were selected for variable inclusion. Subsequently, a stepwise Cox proportional hazards regression model based on maximum likelihood estimation was applied to identify independent factors, with results presented as hazard ratios (HR) and 95% confidence intervals (CI). Two-tailed *p*-values < 0.05 were considered statistically significant.

## 3 Results

### 3.1 Baseline characteristics

A total of 620 CHB patients meeting the criteria were enrolled in this study. To minimize confounding bias, PSM was performed based on gender, age, HBV DNA levels, and LSM. After matching, 247 patients from the CHB group and 247 patients from the MASLD-CHB group were included. The follow-up duration (28.0 [22.0, 36.0] *vs.* 26.5 [21.0, 35.3] months) and Peg-IFN treatment duration (12.4 [10.5, 15.9] *vs.* 11.7 [10.4, 14.5] months) were comparable between the CHB and MASLD-CHB groups (*p* > 0.05). In the CHB group, 61 cases (24.7%) were treated with Peg-IFN alone, while 186 cases (75.3%) received a combination of Peg-IFN and NAs. In the MASLD-CHB group, 72 cases (29.1%) received Peg-IFN monotherapy, and 175 cases (70.9%) received the combination therapy. Additionally, the two groups were comparable in terms of gender, age, HBV DNA, HBsAg, HBeAg positivity rates, and other baseline characteristics. However, the MASLD-CHB group had significantly lower HDL levels than the CHB group (*p* < 0.001). In contrast, the MASLD-CHB group exhibited significantly higher levels of BMI, ALT, AST, GGT, UA, TG, TC, LDL, WBC, NEUT, RBC, HGB, and PLT (**Table 1**).

**Table 1:** Baseline characteristics of the study population.

Variables	CHB group	MASLD-CHB group	*t/Z/χ^2^*	*p* value
	(n = 247)	(n = 247)		
Gender			0	1
Male	208 (84.2%)	208 (84.2%)		
Female	39 (15.8%)	39 (15.8%)		
Age, yr	40 (35, 47)	39 (35, 46)	-0.790	0.429
BMI (kg/m^2^)	24.10 ± 3.61	27.21 ± 3.47	-8.784	<0.001
HBV DNA (log_10_ IU/mL)	1.30 (0, 3.23)	1.30 (1.00, 2.75)	-0.376	0.707
HBV DNA, > 20 IU/mL	114 (46.2%)	110 (44.5%)	0.131	0.718
HBsAg (log_10_ IU/mL)	3.07 (2.56, 3.49)	3.08 (2.09, 3.55)	-0.638	0.524
HBeAg-positive	81 (32.8%)	83 (33.6%)	0.037	0.848
ALT (U/L)	25.75 (17.25, 43.32)	33.30 (21.00, 56.00)	-3.586	<0.001
AST (U/L)	22.00 (18.00, 32.05)	24.00 (19.20, 35.60)	-2.246	0.025
ALB (g/L)	46.96 ± 4.24	47.58 ± 3.58	-1.739	0.083
TBIL (µmol/L)	15.40 (12.70, 19.30)	15.10 (11.40, 19.40)	-1.362	0.173
GGT (U/L)	23.60 (17.17, 38.00)	31.5 (20.30, 50.00)	-3.935	<0.001
BUN (mmol/L)	4.60 (3.80, 5.30)	4.77 (4.00, 5.30)	-1.240	0.215
Cr (μmol/L)	72.37 ± 12.36	73.06 ± 12.55	-0.616	0.538
UA (μmol/L)	329.50 (279.00, 366.85)	376.00 (324.00, 431.70)	-7.377	<0.001
TG (mmol/L)	1.04 (0.73, 1.37)	1.35 (1.03, 1.86)	-6.905	<0.001
TC (mmol/L)	4.48 ± 0.92	4.66 ± 1.06	-1.969	0.049
LDL (mmol/L)	2.71 (2.12, 3.15)	2.68 (2.12, 3.32)	-0.222	0.824
HDL (mmol/L)	1.29 ± 0.28	1.16 ± 0.28	4.941	<0.001
GLU (mmol/L)	5.49 (5.20, 5.88)	5.53 (5.22, 6.07)	-1.806	0.071
WBC (10^9^/L)	5.32 (4.44, 6.40)	5.81 (5.08, 6.95)	-3.979	<0.001
NEUT (10^9^/L)	3.04 (2.31, 3.59)	3.25 (2.63, 3.99)	-2.781	0.005
RBC (10^12^/L)	5.01 ± 0.44	5.11 ± 0.45	-2.670	0.008
HGB (g/L)	152.78 ± 14.60	156.98 ± 19.04	-2.754	0.006
PLT (10^9^/L)	191.88 ± 54.02	214.70 ± 53.36	-4.724	<0.001
AFP (ng/mL)	3.37 (2.27, 5.64)	3.19 (2.37, 4.62)	-0.698	0.485
FT3 (pmol/L)	5.27 (4.71, 5.66)	5.26 (4.88, 5.70)	-0.077	0.938
FT4 (pmol/L)	16.15 (14.81, 17.87)	16.05 (14.59, 17.24)	-0.745	0.456
TSH (mIU/L)	1.80 (1.28, 2.45)	1.73 (1.19, 2.38)	-0.405	0.685
LSM (kPa)	6.40 (5.10, 9.30)	6.60 (5.30, 9.40)	-1.275	0.202
CAP (dB/m)	230.75 (207.75, 261.00)	270.00 (247.00, 299.80)	-9.823	<0.001
Treatment Regimens			1.245	0.261
Peg-IFN monotherapy	61 (24.7%)	72 (29.1%)		
Combination with NAs	186 (75.3%)	175 (70.9%)		
Peg-IFN therapy duration, m	12.4 (10.5, 15.9)	11.7 (10.4, 14.5)	-0.688	0.491

### 3.2 Virological response

**Figure 2:**
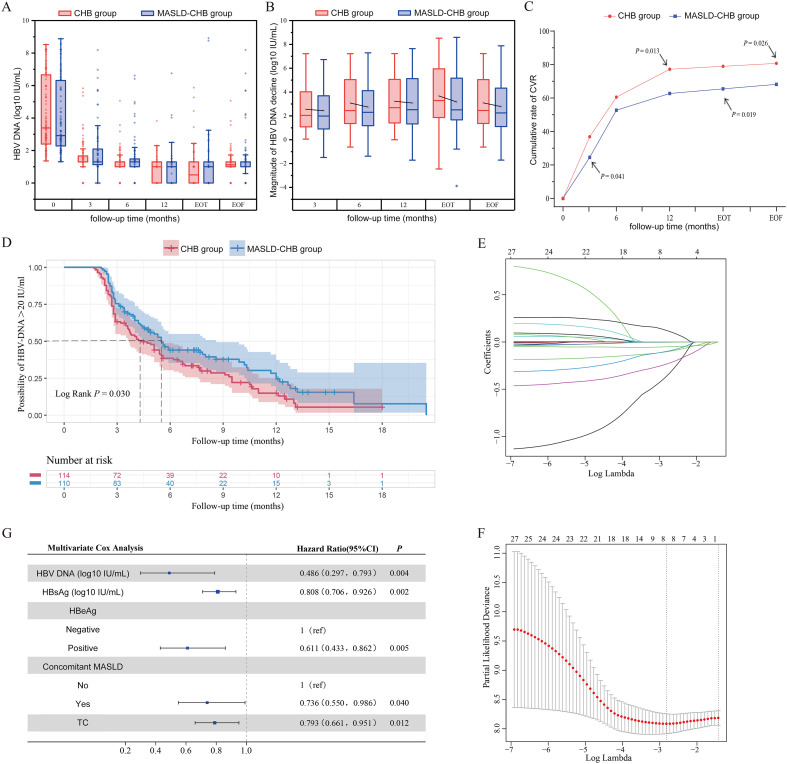
Virological response and CVR-related factors in the CHB and MASLD-CHB groups. **(A)** Changes in HBV DNA levels; **(B)** Changes in the magnitude of HBV DNA decline; **(C)** Cumulative rate of CVR; **(D)** Cumulative risk of persistent HBV DNA positivity (> 20 IU/mL); **(E)** Variable coefficient path diagram from LASSO regression; **(F)** Change in deviance of the log-likelihood as a function of the regularization parameter (Log Lambda); **(G)** Multivariate Cox regression analysis of CVR-related factors and forest plot.

Prior to treatment, 46.2% (n=114) of patients in the CHB group and 44.5% (n=110) of patients in the MASLD-CHB group had HBV DNA levels ≥ 20 IU/mL (**Table 1**). Following Peg-IFN therapy, HBV DNA in the MASLD-CHB group remained higher than in the CHB group at all follow-up points. Furthermore, the reduction in HBV DNA from baseline was significantly smaller in the MASLD-CHB group compared to the CHB group (**Figures 2A, B**), though the differences were not statistically significant. The cumulative rate of CVR was significantly higher in the CHB group compared to the MASLD-CHB group at month 3 (36.84% *vs* 24.54%, *p* = 0.041), month 12 (77.19% *vs* 62.72%, *p* = 0.013), EOT (78.94% *vs* 65.45%, *p* = 0.019), and EOF (80.70% *vs* 68.18%, *p* = 0.026) (**Figure 2C**). Kaplan-Meier analysis revealed that the median time to first CVR was significantly longer in the MASLD-CHB group than in the CHB group (5.5 *vs*. 4.4 months, *p* = 0.030) (**Figure 2D**).

### 3.3 Baseline factors associated with CVR

LASSO regression was employed to identify baseline factors associated with CVR. Based on the minimum λ value, eight potential predictive factors with non-zero coefficients were identified: HBV DNA, HBsAg, HBeAg status, MASLD, TG, HGB, Cr, and HDL (**Figures 2E, F**). Subsequently, multivariate Cox regression analysis was performed to further examine independent baseline factors associated with CVR. The analysis indicated that HBV DNA (HR: 0.486, 95% CI: 0.297-0.793, *p* = 0.004), MASLD (HR: 0.736, 95% CI: 0.550-0.986, *p* = 0.040), HBsAg (HR: 0.808, 95% CI: 0.706-0.926, *p* = 0.002), TG (HR: 0.793, 95% CI: 0.661-0.951, *p* = 0.012), and HBeAg-positive (HR: 0.611, 95% CI: 0.433-0.862, *p* = 0.005) were significantly associated with a reduced likelihood of CVR occurrence (**Figure 2G**).

### 3.4 Serological response

**Figure 3:**
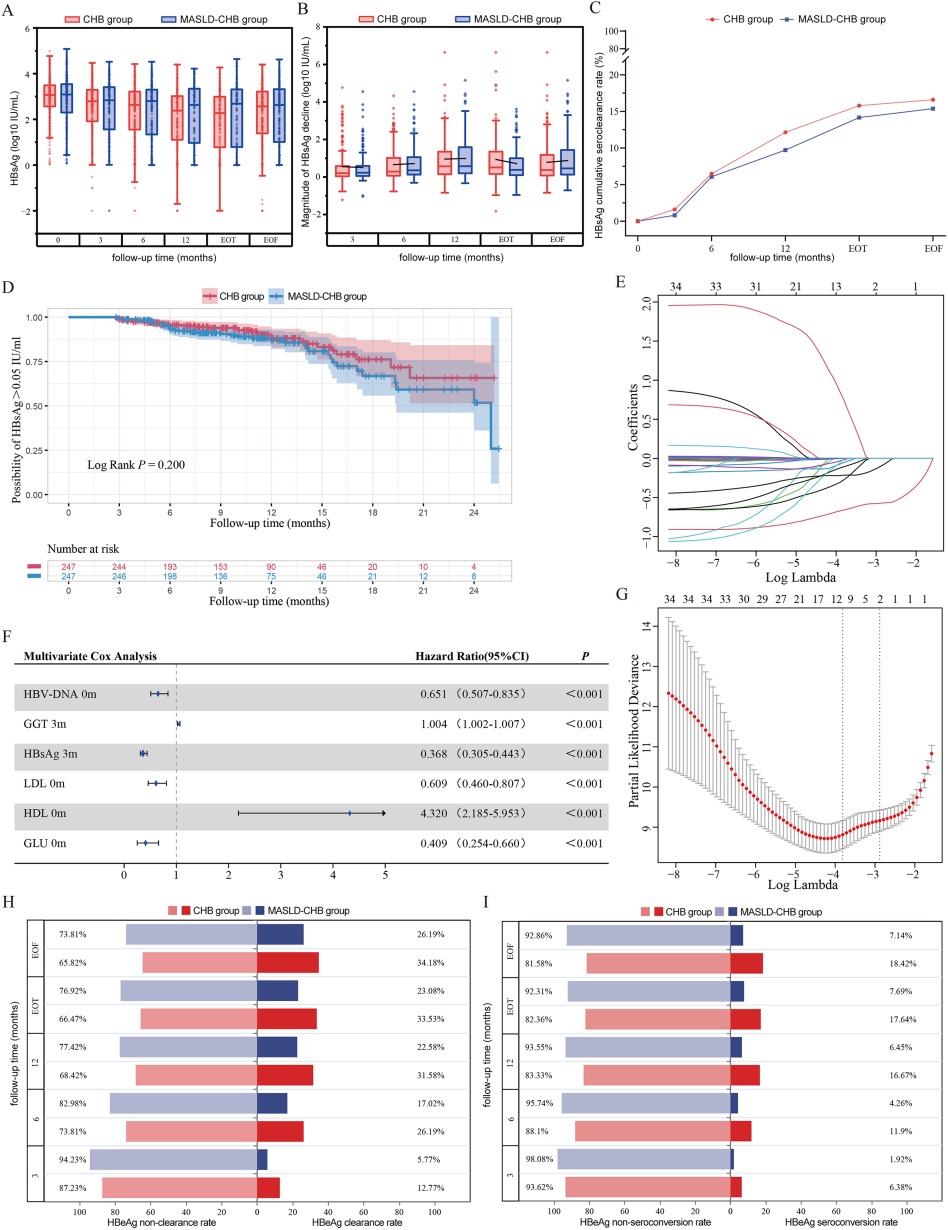
Comparison of factors influencing serological response and HBsAg seroclearance between the CHB and MASLD-CHB groups. **(A)** Comparison of HBsAg level changes; **(B)** Comparison of the magnitude of HBsAg reduction; **(C)** Cumulative rate of HBsAg seroclearance; **(D)** Cumulative risk of sustained HBsAg positivity (> 0.05 IU/mL); **(E)** LASSO regression coefficient path plot for factors associated with HBsAg seroclearance; **(F)** Forest plot of multivariate Cox regression analysis for factors associated with HBsAg seroclearance; **(G)** Plot showing the change in partial likelihood deviation with the regularization parameter (Log Lambda) for factors associated with HBsAg seroclearance; Comparison of **(H)** HBeAg seroclearance rates and **(I)** HBeAg seroconversion rates.

Throughout the entire follow-up period, the HBsAg seroclearance rates in the CHB and MASLD-CHB groups were 16.6% (n = 41) and 15.4% (n = 38), respectively, while the HBsAg seroconversion rates were 10.1% (n = 25) and 8.9% (n = 22), with no statistically significant difference (**Table 1**). During Peg-IFN treatment and the post-treatment follow-up period, both groups showed a gradual increase in HBsAg seroclearance rates as treatment duration extended, although there were no significant differences between the groups at any follow-up time points (**Figure 3C**). Additionally, the HBsAg levels in the MASLD-CHB group were consistently higher than in the CHB group at each follow-up time point, but the difference was not statistically significant (**Figure 3A**). With the extension of treatment duration, both groups exhibited a significant reduction in HBsAg levels at the 6-month, 12-month, EOT, and EOF time points compared to baseline. K-M curve revealed no significant difference in the median time to achieve HBsAg seroclearance between the two groups (Log Rank *p* = 0.200, **Figure 3D**). Across all follow-up time points, the CHB group consistently exhibited higher rates of HBeAg seroclearance and seroconversion than the CHB-MASLD group, although the differences did not reach statistical significance (**Figures 3H, I**).

### 3.5 Factors associated with HBsAg seroclearance

LASSO regression was used to identify factors associated with HBsAg seroclearance. Based on the minimum λ value, 11 potential predictive factors with non-zero coefficients were identified: baseline HBV DNA, ALT at 3 months, AST at 3 months, GGT at 3 months, gender, baseline HBsAg, HBsAg at 3 months, baseline TC, baseline LDL, baseline HDL, and baseline GLU (**Figures 3E–G**). Subsequently, multivariable Cox regression analysis was performed to further identify independent factors associated with HBsAg seroclearance. The analysis revealed that baseline HBV DNA (HR: 0.651, 95% CI: 0.507-0.835, *p* < 0.001), HBsAg at 3 months (HR: 0.368, 95% CI: 0.305-0.443, *p* < 0.001), baseline LDL (HR: 0.609, 95% CI: 0.460-0.807, *p* < 0.001), and baseline GLU (HR: 0.409, 95% CI: 0.254-0.660, *p <* 0.001) were independent protective factors for HBsAg seroclearance. Conversely, GGT at 3 months (HR: 1.004, 95% CI: 1.002-1.007, *p* < 0.001) and baseline HDL (HR: 4.320, 95% CI: 2.185-5.933, *p* < 0.001) were independent risk factors for HBsAg seroclearance (**Figure 3F**).

### 3.6 Dynamic changes in liver function

**Figure 4:**
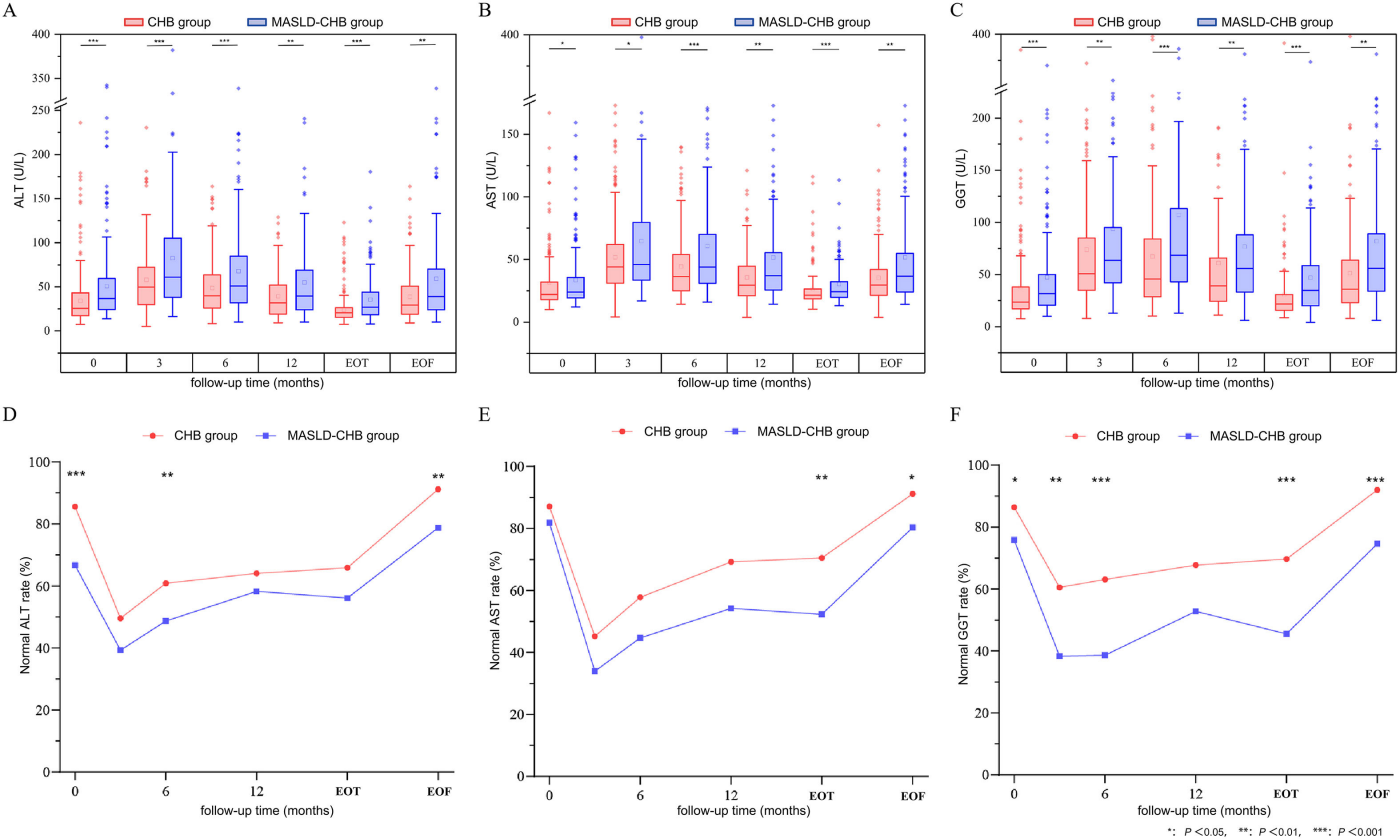
Dynamic changes in liver function between the CHB and MASLD-CHB groups. **(A)** ALT levels and **(D)** normal rates; **(B)** AST levels and **(E)** normal rates; **(C)** GGT levels and **(F)** normal rates. Statistical significance is indicated by asterisks (*p < 0.05, **p < 0.01, and ***p < 0.001).

Comparison of ALT levels between the two groups indicated that the MASLD-CHB group exhibited significantly higher ALT levels than the CHB group at baseline and across all follow-up time points (**Figure 4A**). ALT in both groups was significantly elevated at the 3-month and 6-month follow-up visits compared to baseline (*p* < 0.05). The normal rate of ALT in the MASLD-CHB group was significantly lower than in the CHB group at baseline (66.7% *vs*. 85.6%, *p* < 0.001), 6-month visit (48.7% *vs*. 69.9%, *p* = 0.002), and EOF (78.8% *vs*. 91.2%, *p* = 0.009) (**Figure 4D**). A comparison of AST levels revealed that the MASLD-CHB group had significantly higher AST levels than the CHB group at baseline and all follow-up time points (**Figure 4B**). AST levels in both groups were significantly higher at the 3-month, 6-month, 12-month, and EOT compared to baseline (*p* < 0.05). The normal rate of AST in the MASLD-CHB group was significantly lower than in the CHB group at EOT (52.3% *vs*. 70.5%, *p* = 0.002) and EOF (80.3% *vs*. 91.2%, *p* = 0.019) (**Figure 4E**). A comparison of GGT demonstrated that the MASLD-CHB group had significantly higher GGT levels than the CHB group at all follow-up time points (**Figure 4C**). Within-group comparisons showed that GGT levels were significantly elevated at the 3-month, 6-month, 12-month, and EOT compared to baseline (*p* < 0.05) for both groups. The normal rate of GGT in the MASLD-CHB group was significantly lower than in the CHB group at baseline, 3-month, 6-month, EOT, and EOF (**Figure 4F**).

### 3.7 Dynamic changes in blood lipids

**Figure 5:**
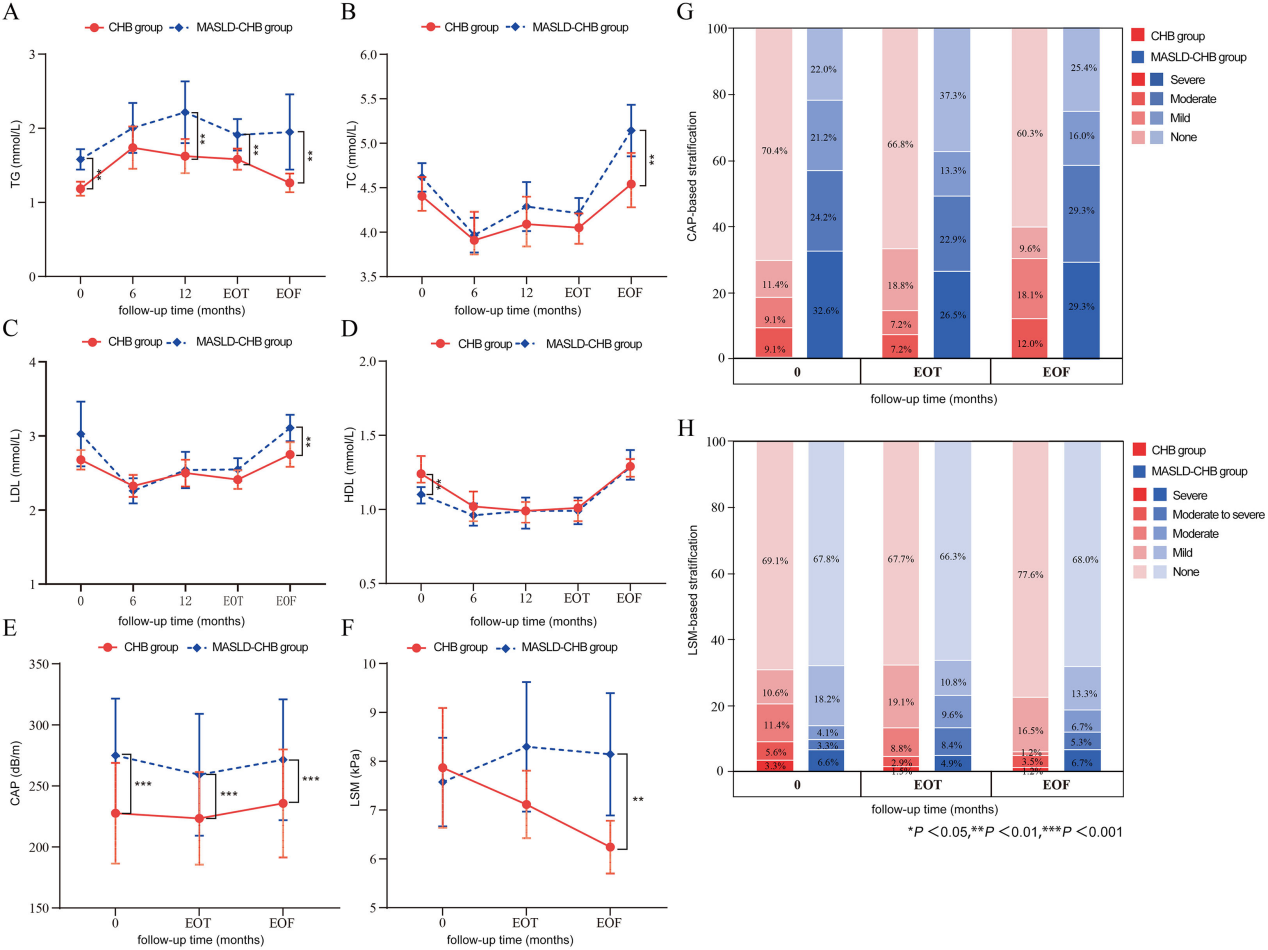
Dynamic changes in serum lipids, hepatic steatosis, and fibrosis in the CHB and MASLD-CHB groups. Comparison of serum **(A)** TG, **(B)** TC, **(C)** LDL, and **(D)** HDL levels; Comparison of **(E)** CAP and **(F)** LSM values; Stacked percentage bar graph of **(G)** CAP and **(H)** LSM-based stratification. Statistical significance is indicated by asterisks (**p < 0.01, and ***p < 0.001).

Comparison of the changes in blood lipid levels between the two groups revealed that the MASLD-CHB group had significantly higher TG levels than the CHB group at month 12, EOT, and EOF. Additionally, both groups exhibited a significant increase in TG levels at month 12 compared to baseline (*p* < 0.01) (**Figure 5A**). The MASLD-CHB group had significantly higher TC levels than the CHB group at EOF, with both groups showing a significant reduction in TC levels at month 6 and EOT compared to baseline (*p* < 0.01) (**Figure 5B**). At EOF, LDL levels were significantly higher in the MASLD-CHB group than in the CHB group (*p* < 0.01), with no significant differences in LDL levels at any follow-up point compared to baseline in either group (**Figure 5C**). No significant differences in HDL levels were observed between the groups at any follow-up point after antiviral treatment, but both groups showed a significant decrease in HDL levels at EOT compared to baseline (*p* < 0.01) (**Figure 5D**).

### 3.8 Dynamic changes in hepatic steatosis and fibrosis

Comparison of hepatic steatosis and fibrosis changes between the two groups following treatment showed that the MASLD-CHB group had significantly higher CAP values and a greater proportion of patients with severe steatosis (CAP > 292 dB/m) than the CHB group (*p* < 0.001). However, no significant differences were observed between the groups at any follow-up point when compared to baseline (**Figures 5E, G**). LSM increased in the MASLD-CHB group after treatment, whereas decreased in the CHB group. At the EOF, the MASLD-CHB group had significantly higher LSM values and a greater proportion of patients with severe fibrosis (LSM > 17.5 kPa) compared to the CHB group (*p* < 0.01). Nevertheless, no significant differences were observed between the groups when compared to baseline (**Figures 5F, H**).

### 3.9 Adverse events and clinical outcomes

During the treatment period, adverse events such as abnormalities in peripheral blood cell counts, fever, musculoskeletal pain, thyroid dysfunction, headache, fatigue, alopecia, and decreased appetite were observed in both groups. The incidence of fatigue was significantly higher in CHB patients with concurrent MASLD than in those with CHB alone (66.4% *vs*. 49.8%, *p* = 0.001). At the EOF, cirrhosis had developed in 5 patients with MASLD-CHB and in 3 patients with CHB alone. No cases of HCC were reported in either group (**Table 2**).

**Table 2:** Incidence of adverse events and clinical outcomes in the two groups.

Variables	CHB group	MASLD-CHB group	*χ^2^*	*p* value
	(n = 247)	(n = 247)		
Adverse events				
Abnormalities in peripheral blood cell counts	127 (51.4%)	109 (44.1%)	2.629	0.102
Fever	174 (70.4%)	190 (76.9%)	2.673	0.097
Musculoskeletal pain	106 (42.9%)	114 (46.2%)	0.524	0.466
Thyroid dysfunction	9 (3.6%)	6 (2.4%)	0.618	0.429
Headache	97 (39.3%)	108 (43.7%)	1.009	0.312
Fatigue	123 (49.8%)	164 (66.4%)	13.978	0.001^*^
Alopecia	52 (21.1%)	62 (25.1%)	1.140	0.281
Decreased appetite	51 (20.6%)	39 (15.8%)	1.956	0.157
Clinical outcomes				
Cirrhosis	3 (1.2%)	5 (2.0%)	0.127	0.720
HCC	0	0	–	–

^*^p < 0.05 was considered statistically significant.

## 4 Discussion

At present, whether concurrent MASLD affects the antiviral efficacy in patients with CHB remains controversial. Previous studies have suggested that concurrent MASLD may exert a negative, positive, or neutral impacts on antiviral treatment outcomes (20–22). Furthermore, prior research has mainly concentrated on NAs therapy (23), while there are limited studies on Peg-IFN-based antiviral regimens. Our study adopts the latest diagnostic criteria, now termed MASLD, to investigate its impact on treatment response in CHB patients receiving Peg-IFN-based therapy and to identify independent factors associated with CVR and HBsAg seroclearance rates. The results indicate that among CHB patients undergoing Peg-IFN-based antiviral treatment, concurrent MASLD may impede the reduction of HBV DNA, significantly delay the time to first CVR, and potentially promote the progression of liver fibrosis. However, MASLD did not significantly affect HBsAg seroclearance in CHB patients.

This study employed a Peg-IFN-based antiviral strategy without distinguishing between specific Peg-IFN protocols. This approach differs from previous studies targeting one or several specific treatment regimens, allowing a more comprehensive and holistic investigation into the overall impact of Peg-IFN-based therapies on HBsAg seroclearance in CHB patients, as well as the effect of concurrent MASLD on antiviral efficacy. A previous prospective study in mainland China found that hepatic steatosis (HS) reduced the efficacy of Peg-IFNα-2a in CHB patients, potentially related to differential expression patterns of HBcAg in liver tissue (20). A retrospective study from Turkey also reported a trend toward reduced virological response to Peg-IFN therapy in CHB patients with HS (24), which is consistent with our findings. Another study involving CHB patients receiving 48 weeks of Peg-IFN therapy showed no difference in HBV DNA suppression rates between MASLD and non-MASLD patients at the EOT. However, 48 weeks after treatment cessation, HBV DNA suppression was lower in MASLD patients, and multivariate analysis identified MASLD as a factor unfavorable to sustained virological response (OR = 0.012, *p* = 0.020) (25). Our study further confirms that MASLD significantly suppresses the occurrence of CVR, and that baseline levels of HBV DNA, HBsAg, TG, as well as HBeAg positivity, are independent factors associated with virological response, also significantly suppressing CVR. Another previous study also found that high baseline ALT levels were correlated with better virological response after 12 months of IFN-α therapy (26). In summary, it appears that among CHB patients receiving Peg-IFN-based treatment, concurrent MASLD may reduce host response to antiviral therapy.

However, some studies have reported conflicting findings, indicating that HS does not affect the efficacy of Peg-IFN therapy at 48 weeks (27, 28). A Turkish study involving 140 CHB patients receiving Peg-IFN-based therapy showed no significant difference in HBV DNA suppression rates between patients with steatosis and those without steatosis (29). These discrepant and even contradictory conclusions across studies may be attributable to small sample sizes—the smallest being 50 patients—which could introduce limitations and inaccuracies in statistical analyses. Variations in HBV DNA detection thresholds may also contribute to the inconsistency; some studies defined virological response as HBV DNA < 500 IU/mL or < 100 IU/mL, whereas our study defined CVR as HBV DNA < 20 IU/mL, enabling a more accurate assessment of viral load. This refined definition assists clinicians in monitoring disease progression in hepatitis patients and evaluating antiviral therapeutic outcomes more precisely. Although multivariate analysis can partially mitigate the effects of certain heterogeneities, it cannot eliminate them entirely. Therefore, this study also employed PSM to control for confounding biases and enhance intergroup comparability. This ensured that patients with CHB alone and those with concurrent MASLD were comparable in key baseline characteristics, allowing a more accurate identification of independent factors associated with CVR. This approach not only improves the generalizability and scientific rigor of the findings—enhancing their credibility—but also provides stronger support for clinical decision-making.

Although no significant differences were observed in HBsAg seroclearance rates, quantitative HBsAg levels, or median time to HBsAg seroclearance at any follow-up time point between the two groups, it is noteworthy that patients with concurrent MASLD consistently exhibited higher quantitative HBsAg levels than those with CHB alone at all follow-up assessments. A study presented at the APASL 2024 conference reported no significant differences in overall HBsAg decline rate or HBsAg seroclearance between CHB patients with and without fatty liver after 48 weeks of Peg-IFN treatment, which is consistent with our results. However, a recent study suggested that Peg-IFN may upregulate ACSL1, increase TG levels, induce HS, and indirectly promote HBsAg seroclearance in CHB patients (30). This finding appears inconsistent with ours, as the current study showed slightly lower HBsAg seroclearance rates in MASLD-CHB patients than in the CHB group (15.4% *vs*. 16.6%, *p* > 0.05) across follow-up visits. The discrepancy may be explained by the enrollment of an advantageous population in the previous study—specifically, CHB patients who had received NAs for at least one year, had HBsAg < 1500 IU/mL, had achieved HBeAg seroconversion, and HBV DNA < 20 IU/mL. In contrast, the present study included patients regularly receiving Peg-IFN therapy who met the diagnostic criteria for CHB, thereby better reflecting real-world clinical conditions. Furthermore, LASSO-Cox regression was used to identify factors influencing HBsAg seroclearance. Although concurrent MASLD was not found to significantly affect seroclearance, baseline levels of HBV DNA, LDL, GLU, and 3-month HBsAg were identified as significant inhibitors of HBsAg seroclearance, whereas baseline HDL and 3-month GGT levels significantly promoted HBsAg seroclearance.

This study used non-invasive LSM to assess the degree of liver fibrosis in CHB patients. After 12 months of Peg-IFN therapy, although most patients had achieved CVR, those with concurrent MASLD still showed potential for fibrosis progression. Previous studies have confirmed that HS can drive fibrosis progression even in patients with undetectable HBV DNA (31). Several cross-sectional studies have reported a positive correlation between steatosis and fibrosis (32, 33). We observed that LSM increased after treatment in the MASLD-CHB group but decreased in the CHB group. At the EOF, LSM values and the proportion of patients with moderate-to-severe fibrosis were significantly higher in the MASLD-CHB group. Additionally, concurrent MASLD was associated with higher levels of ALT, AST, and GGT. Patients in the MASLD-CHB group had significantly higher liver enzyme levels both at baseline and at all follow-up time points, with greater fluctuations during treatment. Moreover, after at least two years of follow-up, cirrhosis developed in five MASLD-CHB patients and three CHB patients.

In this context, this pathogenetic interplay between MASLD and CHB may be partly mediated by steatosis-related immune dysregulation and the formation of a pro-fibrotic hepatic microenvironment. Hepatic steatosis has been shown to modulate immune responses and promote fibrogenic signaling, which may persist despite effective viral suppression and contribute to ongoing liver injury (34). These observations provide a plausible mechanistic basis for the adverse impact of concurrent MASLD on fibrosis progression in CHB patients and support the need for continued attention to steatosis management during antiviral therapy. Beyond its direct effects on liver health, MASLD may also increase patients’ need for medical care (35). Delayed CVR often leads to more frequent follow-ups, additional tests, and prolonged treatment, increasing both dropout risk and financial burden (36). These findings highlight the need to optimize antiviral strategies and address metabolic dysfunction to reduce long-term healthcare burden.

Although the findings of this study have important implications for both clinical practice and future research, several limitations should be acknowledged. First, as the study cohort consisted entirely of outpatients, certain variables such as waist circumference were not fully available; therefore, BMI was used to assess the presence of metabolic syndrome (MetS). Second, this study did not account for dynamic changes in MASLD over time and their potential impact on treatment response in CHB patients. Third, since only patients receiving Peg-IFN-based therapy were included, the sample size and duration of follow-up were limited, which may have reduced the power to detect differences in HBsAg seroclearance and clinical outcomes. Future large-sample, multicenter, transregional, randomized, controlled, prospective studies with long-term follow-up are needed to provide more robust evidence for clinical management and to inform new treatment strategies for CHB patients with MASLD. In addition, integrating animal models, cell-based assays, and multi-omics research may help elucidate the molecular mechanisms underlying the interaction between CHB and MASLD, thereby supplying substantive evidence for clinical decision-making, optimizing management strategies, and supporting national health initiatives.

## 5 Conclusion

Among CHB patients receiving Peg-IFN-based antiviral therapy, concurrent MASLD may impede the reduction of HBV DNA levels, significantly delay the time to first CVR, and potentially accelerate liver fibrosis progression. However, it appears to have no significant effect on quantitative HBsAg levels or seroclearance rates in these patients.

## Data Availability

The original contributions presented in the study are included in the article/supplementary material. Further inquiries can be directed to the corresponding authors.

## References

[1] Shan S Zhao X Jia J . Comprehensive approach to controlling chronic hepatitis B in China. Clin Mol Hepatol. (2024) 30:135–43. doi: 10.3350/cmh.2023.0412, PMID: 38176692 PMC11016498

[2] Howell J Pedrana A Schroeder SE Scott N Aufegger L Atun R . A global investment framework for the elimination of hepatitis B. J Hepatol. (2021) 74:535–49. doi: 10.1016/j.jhep.2020.09.013, PMID: 32971137 PMC7505744

[3] Sung H Ferlay J Siegel RL Laversanne M Soerjomataram I Jemal A . Global cancer statistics 2020: globocan estimates of incidence and mortality worldwide for 36 cancers in 185 countries. CA Cancer J Clin. (2021) 71:209–49. doi: 10.3322/caac.21660, PMID: 33538338

[4] Han SK Baik SK Kim MY . Non-alcoholic fatty liver disease: definition and subtypes. Clin Mol Hepatol. (2023) 29:S5–s16. doi: 10.3350/cmh.2022.0424, PMID: 36577427 PMC10029964

[5] Riazi K Azhari H Charette JH Underwood FE King JA Afshar EE . The prevalence and incidence of nafld worldwide: A systematic review and meta-analysis. Lancet Gastroenterol Hepatol. (2022) 7:851–61. doi: 10.1016/s2468-1253(22)00165-0, PMID: 35798021

[6] Huang SC Liu CJ . Chronic hepatitis B with concurrent metabolic dysfunction-associated fatty liver disease: challenges and perspectives. Clin Mol Hepatol. (2023) 29:320–31. doi: 10.3350/cmh.2022.0422, PMID: 36726053 PMC10121303

[7] MaChado MV Oliveira AG Cortez-Pinto H . Hepatic steatosis in hepatitis B virus infected patients: meta-analysis of risk factors and comparison with hepatitis C infected patients. J Gastroenterol Hepatol. (2011) 26:1361–7. doi: 10.1111/j.1440-1746.2011.06801.x, PMID: 21649726

[8] Zheng Q Zou B Wu Y Yeo Y Wu H Stave CD . Systematic review with meta-analysis: prevalence of hepatic steatosis, fibrosis and associated factors in chronic hepatitis B. Aliment Pharmacol Ther. (2021) 54:1100–9. doi: 10.1111/apt.16595, PMID: 34469587

[9] Rinella ME Lazarus JV Ratziu V Francque SM Sanyal AJ Kanwal F . A multisociety delphi consensus statement on new fatty liver disease nomenclature. J Hepatol. (2023) 79:1542–56. doi: 10.1016/j.jhep.2023.06.003, PMID: 37364790

[10] Jin X Chen YP Yang YD Li YM Zheng L Xu CQ . Association between hepatic steatosis and entecavir treatment failure in chinese patients with chronic hepatitis B. PloS One. (2012) 7:e34198. doi: 10.1371/journal.pone.0034198, PMID: 22479562 PMC3316632

[11] Kim DS Jeon MY Lee HW Kim BK Park JY Kim DY . Influence of hepatic steatosis on the outcomes of patients with chronic hepatitis B treated with entecavir and tenofovir. Clin Mol Hepatol. (2019) 25:283–93. doi: 10.3350/cmh.2018.0054, PMID: 30419649 PMC6759433

[12] Kim SH Cho EJ Jang BO Lee K Choi JK Choi GH . Comparison of biochemical response during antiviral treatment in patients with chronic hepatitis B infection. Liver Int. (2022) 42:320–9. doi: 10.1111/liv.15086, PMID: 34679254

[13] Nathanson V . Revising the declaration of helsinki. Bmj. (2013) 346:f2837. doi: 10.1136/bmj.f2837, PMID: 23657182

[14] European Association for the Study of the Liver (EASL). EASL clinical practice guidelines on the management of hepatitis B virus infection. J Hepatol. (2025) 83:502–83. doi: 10.1016/j.jhep.2025.03.018, PMID: 40348683

[15] European Association for the Study of the Liver (EASL), European Association for the Study of Diabetes (EASD), European Association for the Study of Obesity (EASO). EASL-EASD-EASO clinical practice guidelines on the management of metabolic dysfunction-associated steatotic liver disease (MASLD). J Hepatol. (2024) 81:492–52. doi: 10.1016/j.jhep.2024.04.031, PMID: 38851997

[16] de Lédinghen V Vergniol J Foucher J Merrouche W le Bail B . Non-invasive diagnosis of liver steatosis using controlled attenuation parameter (Cap) and transient elastography. Liver Int. (2012) 32:911–8. doi: 10.1111/j.1478-3231.2012.02820.x, PMID: 22672642

[17] Sugimoto K Moriyasu F Dioguardi Burgio M Vilgrain V Jesper D Strobel D . Us markers and necroinflammation, steatosis, and fibrosis in metabolic dysfunction-associated steatotic liver disease: the ilead study. Radiology. (2024) 312:e233377. doi: 10.1148/radiol.233377, PMID: 39162633

[18] Tang LSY Covert E Wilson E Kottilil S . Chronic hepatitis B infection: A review. Jama. (2018) 319:1802–13. doi: 10.1001/jama.2018.3795, PMID: 29715359

[19] Huang SC Su TH Tseng TC Chen CL Hsu SJ Liu CH . Metabolic dysfunction-associated steatotic liver disease facilitates hepatitis B surface antigen seroclearance and seroconversion. Clin Gastroenterol Hepatol. (2024) 22:581–90.e6. doi: 10.1016/j.cgh.2023.09.040, PMID: 37871842

[20] Liang H Liu Y Jiang X Zheng X Tang J Yang J . Impact of hepatic steatosis on the antiviral effects of peg-ifnα-2a in patients with chronic hepatitis B and the associated mechanism. Gastroenterol Res Pract. (2020) 2020:1794769. doi: 10.1155/2020/1794769, PMID: 32676103 PMC7335408

[21] Hsu YC Yeh ML Wong GL Chen CH Peng CY Buti M . Incidences and determinants of functional cure during entecavir or tenofovir disoproxil fumarate for chronic hepatitis B. J Infect Dis. (2021) 224:1890–9. doi: 10.1093/infdis/jiab241, PMID: 33999179

[22] Li J Le AK Chaung KT Henry L Hoang JK Cheung R . Fatty liver is not independently associated with the rates of complete response to oral antiviral therapy in chronic hepatitis B patients. Liver Int. (2020) 40:1052–61. doi: 10.1111/liv.14415, PMID: 32086988

[23] Ren G Jia K Yin S Guan Y Cong Q Zhu Y . Impact of hepatic steatosis on the efficacy of antiviral treatment for chronic hepatitis B and the establishment of predictive model: A cohort study. Virol J. (2025) 22:30. doi: 10.1186/s12985-025-02642-9, PMID: 39920779 PMC11804069

[24] Ateş F Yalnız M Alan S . Impact of liver steatosis on response to pegylated interferon therapy in patients with chronic hepatitis B. World J Gastroenterol. (2011) 17:4517–22. doi: 10.3748/wjg.v17.i40.4517, PMID: 22110283 PMC3218143

[25] Gong L Liu J Wang J Lou GQ Shi JP . Hepatic steatosis as a predictive factor of antiviral effect of pegylated interferon therapy in patients with hepatitis B. Transplant Proc. (2015) 47:2886–91. doi: 10.1016/j.transproceed.2015.10.023, PMID: 26707308

[26] Li J Zhang X Chen L Zhang Z Zhang J Wang W . Circulating mir-210 and mir-22 combined with alt predict the virological response to interferon-alpha therapy of chb patients. Sci Rep. (2017) 7:15658. doi: 10.1038/s41598-017-15594-0, PMID: 29142236 PMC5688172

[27] Shi JP Lu L Qian JC Ang J Xun YH Guo JC . Impact of liver steatosis on antiviral effects of pegylated interferon-alpha in patients with chronic hepatitis B. Zhonghua Gan Zang Bing Za Zhi. (2012) 20:285–8. doi: 10.3760/cma.j.issn.1007-3418.2012.04.012, PMID: 22964150

[28] Xu L Li P Shi Q Mi Y . Impact of liver steatosis on the curative effect of pegylated interferon-alpha-2a in patients with chronic hepatitis B. Zhonghua Gan Zang Bing Za Zhi. (2015) 23:99–102. doi: 10.3760/cma.j.issn.1007-3418.2015.02.005, PMID: 25880974 PMC12770673

[29] Cindoruk M Karakan T Unal S . Hepatic steatosis has no impact on the outcome of treatment in patients with chronic hepatitis B infection. J Clin Gastroenterol. (2007) 41:513–7. doi: 10.1097/01.mcg.0000225586.78330.60, PMID: 17450036

[30] Wu L Li Z Gao N Deng H Zhao Q Hu Z . Interferon-A Could induce liver steatosis to promote hbsag loss by increasing triglyceride level. Heliyon. (2024) 10:e32730. doi: 10.1016/j.heliyon.2024.e32730, PMID: 38975233 PMC11226829

[31] Mak LY Hui RW Fung J Liu F Wong DK Cheung KS . Diverse effects of hepatic steatosis on fibrosis progression and functional cure in virologically quiescent chronic hepatitis B. J Hepatol. (2020) 73:800–6. doi: 10.1016/j.jhep.2020.05.040, PMID: 32504663

[32] Seto WK Hui RWH Mak LY Fung J Cheung KS Liu KSH . Association between hepatic steatosis, measured by controlled attenuation parameter, and fibrosis burden in chronic hepatitis B. Clin Gastroenterol Hepatol. (2018) 16:575–83.e2. doi: 10.1016/j.cgh.2017.09.044, PMID: 28970146

[33] Choi HSJ Brouwer WP Zanjir WMR de Man RA Feld JJ Hansen BE . Nonalcoholic steatohepatitis is associated with liver-related outcomes and all-cause mortality in chronic hepatitis B. Hepatology. (2020) 71:539–48. doi: 10.1002/hep.30857, PMID: 31309589

[34] Zhang S Mak LY Yuen MF Seto WK . Mechanisms of hepatocellular carcinoma and cirrhosis development in concurrent steatotic liver disease and chronic hepatitis B. Clin Mol Hepatol. (2025) 31:S182–s95. doi: 10.3350/cmh.2024.0837, PMID: 39568126 PMC11925439

[35] Allen AM Lazarus JV Younossi ZM . Healthcare and socioeconomic costs of nafld: A global framework to navigate the uncertainties. J Hepatol. (2023) 79:209–17. doi: 10.1016/j.jhep.2023.01.026, PMID: 36740046 PMC10293095

[36] Toy M Hutton D Jia J So S . Costs and health impact of delayed implementation of a national hepatitis B treatment program in China. J Glob Health. (2022) 12:4043. doi: 10.7189/jogh.12.04043, PMID: 35796158 PMC9260492

